# Good manufacturing practice in low‐ and middle‐income countries: Challenges and solutions for compliance

**DOI:** 10.1002/puh2.158

**Published:** 2024-01-30

**Authors:** Pelden Chejor, Thinley Dorji, Ngawang Dema, Andrew Stafford

**Affiliations:** ^1^ Centre for Research in Aged Care Edith Cowan University Joondalup Western Australia Australia; ^2^ Department of Internal Medicine Central Regional Referral Hospital Gelegphu Bhutan; ^3^ Bhutan Food and Drug Authority, Royal Government of Bhutan Thimphu Bhutan; ^4^ Curtin Medical School enAble Institute Faculty of Health Sciences Curtin University Bentley Western Australia Australia

**Keywords:** inspections, medicines, pharmaceuticals, quality, safe, substandard

## Abstract

Approximately 2 billion people globally have limited access to essential medicines, particularly those living in low‐ and middle‐income countries (LMICs). In addition, there is a higher prevalence of substandard and counterfeit medicines in LMICs, which may cause antimicrobial resistance and therapeutic failure. Good manufacturing practice (GMP) is key to ensuring access to high‐quality, safe and effective medicines, including active pharmaceutical ingredients and excipients used for manufacturing finished dosage forms. Compliance with GMP standards saves resources for both manufacturers (e.g. avoiding expensive product recalls) and governments (e.g. minimising compliance inspections). Concerted collaborations among different countries to harmonise GMP standards and procedures have occurred, and the World Health Organisation (WHO) GMP is commonly used in LMICs. However, barriers to GMP compliance remain apparent, particularly in LMICs. Pharmaceutical manufacturers in LMICs face several barriers such as inadequate resources and infrastructure, inefficient or weak regulation, a shortage of skilled people and limited training opportunities for manufacturing personnel. Although national regulatory authorities (NRAs) have been established in LMICs, most NRAs are not fully independent and are placed within the health department. NRAs in LMICs are not able to fully utilise international and regional reliance mechanisms and collaborations. Therefore, both NRAs and pharmaceutical manufacturers must be adequately supported through resources, infrastructure and capacity‐building opportunities. NRAs must be suitably empowered with assured functional independence. A comprehensive guideline for managing conflicts of interest among pharmaceutical manufacturers, procurement agencies and NRAs is needed. NRAs must liaise with other NRAs, participate in joint GMP inspections and leverage the expertise of regional or international collaborations. Pharmaceutical manufacturers in LMICs can leverage WHO GMP standards to improve GMP compliance and expand their market. However, as these solutions may have cost implications, prioritising key gaps for GMP compliance and optimisation of available resources are essential.

## INTRODUCTION

Approximately 2 billion people globally lack access to essential medicines, particularly in low‐ and middle‐income countries (LMICs) due to inadequate infrastructure, human resources, regulatory provisions and low manufacturing capacity [1]. It is estimated that 10% of all medical products in LMICs are substandard or falsified, resulting in antimicrobial resistance, therapeutic failure and toxicity [2]. Furthermore, 13.6% of essential medicines in LMICs, mostly antibiotics and antimalarials, were found to be counterfeited [3]. One of the targets of the United Nations Sustainable Development Goal 3 ‘Good Health and Wellbeing’ is to achieve access to safe, effective, quality and affordable essential medicines and vaccines for every individual [4]. Pharmaceutical manufacturers play a crucial role in achieving this goal by producing quality medicines in conformance with good manufacturing practice (GMP) standards.

GMP ensures the consistent production of quality, safe and effective medicines appropriate for their intended use and in compliance with regulatory requirements [5]. It covers all aspects of medicines production, including raw materials, personnel involved, premises, equipment, quality control and testing. It is designed to minimise inherent risks (e.g. contamination, mislabelling) that cannot be eliminated by testing the final product [5]. GMP ensures the authenticity of active pharmaceutical ingredients and excipients as an adulteration of these substances can cause significant harm. For example, the substitution of glycerol with diethylene glycol in paediatric cough syrups was associated with numerous deaths in Gambia [6]. GMP prevents expensive product recalls, hefty fines and compensations and cuts government expenditures on enforcement.

However, compliance with GMP varies across countries or regions due to differences in resources, infrastructure and regulatory capacities. Pharmaceutical manufacturers in LMICs face several challenges in complying with GMP and consequently often operate below the required standards [7, 8]. Documenting the challenges facing LMICs in implementing GMP standards will help disseminate information and develop mitigation strategies to improve compliance. However, there is limited literature on barriers and solutions for GMP compliance in LMICs. This paper describes the current state of GMP, issues affecting GMP in LMICs and provides potential solutions for improving compliance.

## THE CURRENT STATE OF GMP WORLDWIDE

GMP standards vary depending on the country or the region where medicine is being manufactured or distributed. Commonly used GMP guidelines include the European Union (EU) GMP, the Pharmaceutical Inspection Cooperation/Scheme (PIC/S) GMP and the World Health Organisation (WHO) GMP, with the latter primarily being used in LMICs [9]. GMP compliance is a prerequisite for medical product registration in Thailand and Korea, unlike other Southeast Asian countries. The type of GMP standard used to assess GMP compliance has implications for market authorisation. For example, medicines manufactured in some LMICs may not be recognised at par with other GMP standards (e.g. EU GMP) because GMP requirements may be different (more stringent) in the EU than in LMICs. For this reason, non‐EU European countries such as Norway, Turkey and Switzerland follow EU GMP as it makes it easier for them to export medicines to Europe [9]. Other factors such as business tariffs, taxes and trade agreements may also impede pharmaceutical manufacturers in LMICs from breaking into European markets.

A mutual recognition agreement between the countries can help overcome GMP differences across countries. For example, medicines manufactured anywhere in EU countries are recognised as equivalent to EU GMP standards. This is because the EU relies on GMP inspections performed by any of the EU national regulatory authorities (NRAs) as they have common inspection procedures and training and qualifications for inspectors [10]. Several associations have been initiated in LMICs to promote reliance and information sharing between NRAs such as the South–East Asia Regulatory Network (SEARN) and the Association of Southeast Asian Nations (ASEAN) [10]. The Pan American Health Organization (PAHO) and African Medicines Agency (AMA) are other reliance‐focused initiatives to focus on improving access to medicines in their respective regions.

## BARRIERS TO GMP COMPLIANCE

Adequate resources, infrastructure and skilled people are critical for both manufacturing and regulation to ensure access to quality medicines. Pharmaceutical manufacturers in LMICs face several challenges in implementing GMP such as resource constraints, poor infrastructure, weak regulatory and enforcement systems, inadequate technical capacity and limited GMP training [7, 8]. Evidence from 26 sub‐Saharan African countries shows weak regulations, inadequate resources and a lack of investment and incentives to promote local manufacturing as hindrances to GMP compliance [11]. A case study from Nepal reports that low regulatory capacity, inadequate training, financial constraints and lack of investment affected GMP compliance [12].

Similarly, NRAs do not have adequate resources and capacity to assess and monitor manufacturers, which may impact the timely approval of drugs. Inadequate assessment for GMP compliance may breed complacency among the manufacturers, which may result in the production of substandard drugs [8]. Enforcement and manufacturing personnel do not receive periodic training on GMP due to a lack of budget.

Most NRAs function within the Ministry of Health or a health department risking possible conflict of interest between NRAs and procurement agencies. Although they both work towards protecting public health, inherent conflicts of interest may exist along the line of their duty [13]. Procurement agencies might focus on making medicines available, whereas NRAs are responsible for ensuring quality medicines, so situations where supply is limited yet quality is sub‐par may create tension between these agencies. Conflicts of interest can be pervasive and may influence the regulation of medicines [13]. Governments slashing the prices of medicines to achieve universal health coverage (possibly due to conflict of interest) may influence the quality of medicines. For example, a manufacturer in Indonesia ceased production as the government introduced a ceiling on the prices of medicines, which resulted in drug shortages in that country [14].

Several international collaborations (e.g. PIC/S) have been established to safeguard the quality and safety of medicines. However, many LMICs are not members of these partnerships and do not have access to benefits from such collaborations. This is rather opposite to the requirements of smaller and poorer countries dealing with a higher prevalence of substandard and falsified medicines [2, 3]. Further, regional collaborations (e.g. SEARN and ASEAN) may not consider country‐specific barriers such as skill shortages and infrastructure necessary for GMP compliance [10]. Individual countries must work on improving GMP and access to medicines for their population. Similarly, SEARN and the AMA are relatively new and their impact on GMP and access to quality medicines is yet to be assessed.

## SOLUTIONS FOR GMP COMPLIANCE

GMP compliance is key to ensuring access to quality, safe and effective medicines. Governments must support pharmaceutical manufacturers and NRAs, ensure the functional independence of NRAs and continue collaborations among stakeholders and international partnerships to ensure GMP compliance and improve access to medicine. A key function of this support is liaison with external experts for GMP training of regulatory and manufacturing personnel. Second, periodic training of manufacturing personnel and NRA inspectors on GMP may be made mandatory. Embedding materials regarding GMP compliance within pharmacy courses may complement this training and provide additional human resources within the pharmacist workforce. Third, the government may provide tax exemptions for materials imported for manufacturing medicines, which may further encourage manufacturers to invest in training their staff and improving GMP compliance. Other incentives, such as low interest rate loans and free land for setting up manufacturing firms, creating a conducive environment for manufacturing and developing effective drug policies, are critical. Strengthening the functional maturity of NRAs through better resourcing, infrastructure and political support is essential.

Although GMP is important for global health, it must not barricade manufacturers from using innovative ideas and technologies. Governments must support and encourage manufacturers to use new technologies and ideas by providing tax exemptions on materials imported for manufacturing medicines and via capacity building. NRAs also need to function independently of the procurement agencies to prevent possible conflicts of interest. For this, there is a need for a guideline to assess, monitor and manage possible conflicts of interest between manufacturers, procurement agencies and NRAs which is currently lacking in LMICs [13].

The ongoing collaborations among NRAs and international/regional partnerships must continue. Resource‐constrained countries can leverage regional partnerships and reliance mechanisms for capacity development and technology transfer [10]. In particular, one country's NRA should liaise with other NRAs in their region for joint GMP inspections and leverage the resources provided by such collaborations. However, there should be strong enforcement mechanisms and monitoring systems for ensuring GMP compliance. LMICs may leverage WHO GMP compliance requirements to both improve medicine safety and expand the market of local pharmaceutical manufacturers. For example, certain manufacturers in Nigeria have achieved WHO GMP certification, leading to their products being marketed internationally [15]. Although these solutions may have initial resourcing implications (e.g. cost and staff recruitment), a staged, product‐focused approach may facilitate the process.

## CONCLUSION

GMP is essential for ensuring access to quality, safe and effective medicines. Pharmaceutical manufacturers in LMICs face several challenges in implementing GMP standards. Adequate infrastructure and capacity‐building opportunities must be provided to manufacturers and NRAs, although these may have cost implications. Resource‐constrained countries must leverage WHO GMP standards to improve GMP compliance and enhance regional and international collaborations for knowledge and technology transfer.

## AUTHOR CONTRIBUTIONS

*Writing of draft; editing and review; proofreading*: Pelden Chejor. *Editing and review; proofreading*: Thinley Dorji. *Editing; review and proofreading*: Ngawang Dema and Andrew Stafford.

## CONFLICT OF INTEREST STATEMENT

Thinley Dorji is an Editorial Board member of Public Health Challenges. He was blinded from peer review and all processes leading to the acceptance of this article for publication.

## FUNDING INFORMATION

The authors have received no funding for the development of this paper.

## References

[1] World Health Organization (WHO) . Ten years in public health 2007–2017. In: Report by Dr Margaret Chan, Director‐General—Access to Medicines: Making Market Forces Serve the Poor. WHO; 2017. Accessed August 24, 2023. https://apps.who.int/iris/bitstream/handle/10665/255355/9789241512442‐eng.pdf?sequence=1&isAllowed=y

[2] World Health Organization (WHO) . Substandard and Falsified Medical Products – Key Facts. WHO; 2023. Accessed August 24, 2023. https://www.who.int/news‐room/fact‐sheets/detail/substandard‐and‐falsified‐medical‐products

[3] Ozawa S , Evans DR , Bessias S , et al. Prevalence and estimated economic burden of substandard and falsified medicines in low‐ and middle‐income countries: a systematic review and meta‐analysis. JAMA Netw Open. 2018;1(4):e181662. doi:10.1001/jamanetworkopen.2018.1662 30646106 PMC6324280

[4] United Nations . Sustainable Development Goals: 17 Goals to Transform Our World. United Nations; 2023. Accessed March 3, 2023. https://www.un.org/sustainabledevelopment/health/

[5] WHO . Questions and Answers. Medicines: Good Manufacturing Practices. WHO; 2015. Accessed January 31, 2023. https://www.who.int/news‐room/questions‐and‐answers/item/medicines‐good‐manufacturing‐processes

[6] Chopra H , Attia MS , Badshah SF , Dhama K , Emran TB . Cough syrups: silent killer of Gambian children. Int J Surg. 2023;109(2):150‐152. doi:10.1097/js9.0000000000000057 36799833 PMC10389612

[7] Tauqeer F , Myhr K , Gopinathan U . Institutional barriers and enablers to implementing and complying with internationally accepted quality standards in the local pharmaceutical industry of Pakistan: a qualitative study. Health Policy Plan. 2019;34(6):440‐449. doi:10.1093/heapol/czz054 31302684 PMC6736431

[8] Lubowa N , Ekeocha Z , Byrn S , Clase K . Pharmaceutical industry in Uganda: a review of the common GMP non‐conformances during regulatory inspections. In: BIRS Africa Technical Report Papers [Paper 10]. Purdue University; 2021. doi:10.5703/1288284317442

[9] Parry D . World good manufacturing practices. In: Bunn PG , ed. In: Good Manufacturing Practices for Pharmaceuticals. 7th ed. CRC Press; 2018:343‐351.

[10] Gostin LO , Wood AJ , Cuff PA . Regulating medicines in a globalized world with increased recognition and reliance among regulators: a National Academies report. JAMA. 2020;324(2):145‐146. doi:10.1001/jama.2019.21793 32134427

[11] WHO . Assessment of Medicines Regulatory Systems in 26 Sub‐Saharan African Countries: An Overview of Findings from 26 Assessment Reports. World Health Organization; 2010.

[12] Brhlikova P , Harper I , Subedi M , Bhattarai S , Rawal N , Pollock AM . Aid conditionalities, International Good Manufacturing Practice Standards and Local Production Rights: a case study of local production in Nepal. Global Health. 2015;11:25. doi:10.1186/s12992-015-0110-3 26072308 PMC4470019

[13] Khan M , Rahman‐Shepherd A , Bory S , et al. How conflicts of interest hinder effective regulation of healthcare: an analysis of antimicrobial use regulation in Cambodia, Indonesia and Pakistan. BMJ Glob Health. 2022;7(5):e008596. doi:10.1136/bmjgh-2022-008596 PMC9121421 35589155

[14] Hasnida A , Kok MO , Pisani E . Challenges in maintaining medicine quality while aiming for universal health coverage: a qualitative analysis from Indonesia. BMJ Glob Health. 2021;6(3):e003663. doi:10.1136/bmjgh-2020-003663. Suppl. PMC8166595 34049935

[15] Anyakora C , Ekwunife O , Alozie F , et al. Cost–benefit of investment on quality in pharmaceutical manufacturing: WHO GMP pre‐ and post‐certification of a Nigerian pharmaceutical manufacturer. BMC Health Serv Res. 2017;17:665. doi:10.1186/s12913-017-2610-8 28923044 PMC5604295

